# Sulforaphane induces cell cycle arrest by protecting RB-E2F-1 complex in epithelial ovarian cancer cells

**DOI:** 10.1186/1476-4598-9-47

**Published:** 2010-03-02

**Authors:** Christopher S Bryant, Sanjeev Kumar, Sreedhar Chamala, Jay Shah, Jagannath Pal, Mahdi Haider, Shelly Seward, Aamer M Qazi, Robert Morris, Assaad Semaan, Masood A Shammas, Christopher Steffes, Ravindra B Potti, Madhu Prasad, Donald W Weaver, Ramesh B Batchu

**Affiliations:** 1 Department of Surgery, Wayne State University, 4100 John R Street, Detroit, MI. 48201, USA; 2 Dept of Ob/Gyn, Wayne State University, 4100 John R Street, Detroit, MI. 48201, USA; 3 Karmanos Cancer Institute, Wayne State University, 4100 John R Street, Detroit, MI. 48201, USA

## Abstract

**Background:**

Sulforaphane (SFN), an isothiocyanate phytochemical present predominantly in cruciferous vegetables such as brussels sprout and broccoli, is considered a promising chemo-preventive agent against cancer. In-vitro exposure to SFN appears to result in the induction of apoptosis and cell-cycle arrest in a variety of tumor types. However, the molecular mechanisms leading to the inhibition of cell cycle progression by SFN are poorly understood in epithelial ovarian cancer cells (EOC). The aim of this study is to understand the signaling mechanisms through which SFN influences the cell growth and proliferation in EOC.

**Results:**

SFN at concentrations of 5 - 20 μM induced a dose-dependent suppression of growth in cell lines MDAH 2774 and SkOV-3 with an IC50 of ~8 μM after a 3 day exposure. Combination treatment with chemotherapeutic agent, paclitaxel, resulted in additive growth suppression. SFN at ~8 μM decreased growth by 40% and 20% on day 1 in MDAH 2774 and SkOV-3, respectively. Cells treated with cytotoxic concentrations of SFN have reduced cell migration and increased apoptotic cell death via an increase in Bak/Bcl-2 ratio and cleavage of procaspase-9 and poly (ADP-ribose)-polymerase (PARP). Gene expression profile analysis of cell cycle regulated proteins demonstrated increased levels of tumor suppressor retinoblastoma protein (RB) and decreased levels of E2F-1 transcription factor. SFN treatment resulted in G1 cell cycle arrest through down modulation of RB phosphorylation and by protecting the RB-E2F-1 complex.

**Conclusions:**

SFN induces growth arrest and apoptosis in EOC cells. Inhibition of retinoblastoma (RB) phosphorylation and reduction in levels of free E2F-1 appear to play an important role in EOC growth arrest.

## Background

Epithelial ovarian cancer is the leading cause of mortality from gynecological malignancies, often undetectable in early stage. The difficulty of detecting the disease in its early stages and the propensity of ovarian cancer cells to develop resistance to known chemotherapeutic treatments dramatically decreases the overall survival [1,2]. Cytoreductive surgery followed by adjuvant combination chemotherapy is the standard treatment for advanced stage disease. However, despite these interventions, long-term survival rates remain low underscoring the need for new effective drugs or drug combinations with tolerable side effects [3,4].

Epidemiological observations indicate that cruciferous vegetables such as broccoli, cabbage, cauliflower and brussels sprouts have been shown to offer protection against various cancers [5,6]. Phytochemicals such as glucosinolates are abundant in these vegetables that are converted into isothiocyanates such as sulforaphane (SFN) [6-8].

Recently it has been shown that SFN inhibits the growth of the epithelial ovarian cancer cell (EOC) line SkOV-3 by down-regulating AKT activity [9]. Related compounds such as synthetic isothiocyanate derivative ethyl 4-isothiocyanatobutanoate (E-4IB) and phenylisothiocyanates have been shown to induce apoptosis in A2780 and OVCAR-3 EOC cell lines [10-13]. Cytotoxic activity has also been demonstrated in SkOV-3 after treatment with indole-3-ethyl isothiocyanate (NB7 M) resulted in the inhibition of PIK3/AKT pathway [14]. Although upstream mechanism of action at the cell surface has been linked to the inhibition of PIK-3 and AKT pathways in earlier studies, the various down stream signaling pathways responsible for SFN activity are not thoroughly understood.

The retinoblastoma protein (RB) is a well-known regulator of G1-S phase cell cycle transition [15]. Negative regulation of the cell cycle is due to the ability of active, under phosphorylated RB to bind the transcription factor E2F-1 and repress transcription required for S phase progression [15,16]. Here we show that SFN enhances the RB-E2F-1 interaction in MDAH-2774 ovarian cancer cell line linking for the first time the upstream AKT inhibition to the downstream blocking of cell cycle by activating RB protein. Further we show the inhibition of invasive ability of cells and reduced migration. Also we document the increased activity of caspases-9 and also cleavage of PARP protein indicating inhibition of cell proliferation and induction of cellular apoptosis.

## Materials and methods

### Reagents and antibodies

Sulforaphane (Sigma chemicals, St. Louis, MO) was dissolved in DMSO and Stock solutions were freshly prepared and added to the cell cultures to obtain the indicated final concentrations. The DMSO concentration was used at 0.01% and the same concentration was used as a vehicle. DMSO alone (0.01%) was found to have no significant effect on cellular function. Cell proliferation assays were conducted using Cell Counting Kit-8 (CCK-8) (Dojindo, Gaithersburg, MD). Cell cycle analysis was conducted using Cell Cycle Phase Determination Kit (Cayman Chemical Company, Ann Arbor, MI). Human fibroblasts were similarly treated as cancer cells to display differential cytotoxicity at any given dose. Retinoblastoma and E2F-1 antibodies were purchased from Millipore (Danvers, MA). Cyclins, CDKs, poly (ADP-ribose) polymerase (PARP) and β actin antibodies were purchased from Santa Cruz Biotechnology, (Santa Cruz, CA).

### Cell lines and culture

Ovarian cancer cell lines, MDAH 2774 and SkOV-3 (American Type Culture Collection, Manassas, VA) were propagated in McCoy's 5A medium supplemented with 10% fetal bovine serum, 2 mM L-glutamine, 100 units/ml penicillin, and 100 mg/ml streptomycin (Thermo Fisher Scientific, Pittsburgh, PA). Cells were cultured in a humidified atmosphere with 5% CO_2 _at 37°C. Trypsin (0.25%)/EDTA solution was used to detach the cells from the culture flask for passing the cells.

### Cell proliferation assays

Standard prototype growth curves and number of viable cells were determined for each cell line (treated and control groups) in triplicate experiments according to the CCK-8 (Dojindo, Gaithersburg, MD) manufactures' instructions. Growth curves were plotted as a percentage of the value of DMSO-treated controls minus the value of untreated cells on day 0. Day 3 values were considered for the determination of the 50% cell proliferation inhibition (IC50) for a given treatment. In some cases parallel manual count was also performed with trypan blue and counting by exclusion method using a Hemocytometer. The findings confirmed CCK-8 assay results.

### Analysis of apoptotic cells

Apoptotic cells were analyzed by using Annexin V FITC apoptosis detection kit (Calbiochem, Gibbstown, NJ) according to the manufacturers instructions. The DNA content of the cells was analyzed by flow cytometer and sub G1 population was considered to represent apoptotic cells. For fluorescent microscopic image analysis of apoptotic fraction of the cells, treated and control (1 × 10^6 ^cells/ml) were mixed with annexin V-biotin and medium-binding reagent, and incubated in the dark for 15 min at room temperature. Cells were then centrifuged and medium was replaced with 1× Binding Buffer containing FITC-streptavidin. Propidium iodide was added to discriminate early apoptotic cells from late apoptotic or necrotic cells. A portion of cell suspension (50 μl) was placed onto a glass slide, covered with a cover slip, and viewed immediately using a fluorescence microscope equipped with FITC (green) and propidium iodide (red) Filters (Zeiss, AXio CamMRm Observer. A1).

### Cell cycle phase determination

MDAH-2774 cells were seeded at 10^6 ^cells in 10 cm dishes and the culture medium changed to serum-free medium for 24 h to facilitate cell cycle synchronization. Cell cycle analysis was conducted using Cell cycle phase determination kit according to the manufacturer's instructions (Cayman chemical company, Ann Arbor, MI). Samples were analyzed in FL2 channel of flow cytometer with a 488 nm excitation laser.

### Western blotting and Immunoprecipitations

These assays were performed according to our earlier publication[17]. Antibody reactions were visualized using enhanced chemiluminescence western blotting detection reagents (Amersham Pharmacia, Uppsala, Sweden).

### Gene Expression Profiling (GEP)

Briefly, PANC-1 cells untreated or treated with 15 μM ritonavir for 48 h, were harvested and total RNA was isolated utilizing an RNeasy kit (Qiagen Inc., Valencia, CA) as described by the manufacturer. Total RNA was sent to MOgene company (MOgene, LC, Saint Louis, MO) for GEP analysis.

## Results

### SFN induces growth arrest in ovarian cancer cell lines

**Figure 1:**
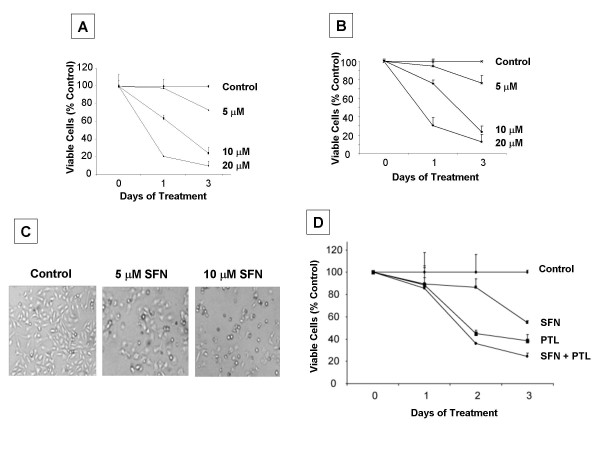
**Effect of sulforaphane (SFN) on the growth of ovarian cancer cell lines with and without paclitaxel**. Cells were cultured for indicated time intervals with various concentrations of the SFN. Cell growth was assessed by CCK-8 cell proliferation assay method. A and B are MDAH-2774 and SkOV-3 cell lines respectively. Graph is expressed as a percentage of control (DMSO treated cells) and represents the mean of triplicate cultures. C. Phase contrast pictures taken at 100× magnification after indicated concentrations of drug treatment of MDAH-2774 cells. D. Paclitaxel mediated cytotoxicity with or without SFN on MDAH-2774 cells. SFN: Sulforaphane; PTL: Paclitaxel.

To determine if SFN had potential to induce growth arrest in ovarian cancer cells, we conducted cell proliferation assays using two human ovarian cancer cell lines, MDAH-2774 and SkOV-3. Cells were grown as sub-confluent monolayer cultures and propagated under standard conditions. Cell lines were treated with serial dilutions of SFN dissolved in DMSO. Human fibroblasts were similarly treated to display differential cytotoxicity (data not shown). SFN treatment resulted in a concentration-dependent inhibition of the proliferation of MDAH 2774 (Fig 1A) and SkOV-3 (Fig 1B) with an IC50 of ~8 μM. The extent of growth inhibition increased as a function of time in all the tested doses ranging from 5 to 20 μM. The extent of cell death with SFN increased as a function of dosage in 48 h as observed by phase contract microscopy in MDAH-2774 cell line (Fig. 1C). Since chemotherapy of ovarian cancer usually consists of paclitaxel alone or in combination with platinum based drugs, we further evaluated the influence of SFN combined with paclitaxel in treating MDAH-2774 cell line. Cells treated with paclitaxel with or without SFN and viability was assessed for 3 days. As shown in fig. 1D, 8 μM SFN which is IC50 for MDAH-2774 or 2 μM paclitaxel alone produced ~40% and ~55% cell death, respectively; however combination treatment resulted in ~70% cell death.

### SFN induces apoptosis MDAH-2774 cells

**Figure 2:**
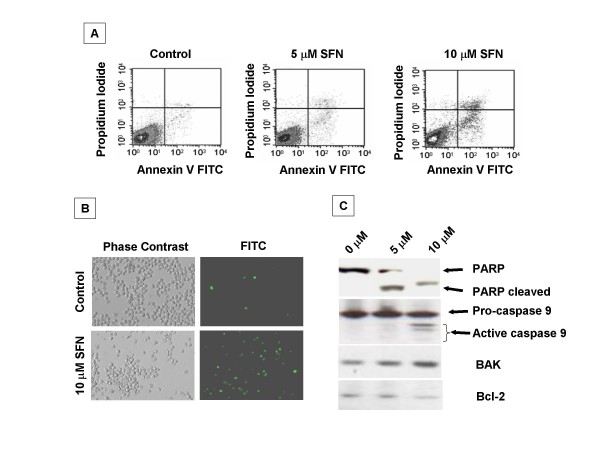
**Analysis apoptotic cells with SFN treatment**. A. Apoptotic cells were analyzed by using Annexin V FITC apoptosis detection kit (Calbiochem, Gibbstown, NJ). Control and SFN treated MDAH-2774 cells (1 × 10^5 ^cells) were fixed, stained both with Annexin V FITC and with propidium iodide (20 μg/ml) and were processed for flow cytometric analysis. B. Cells were mixed with annexin V-biotin, incubated for 15 min at room temperature, and treated with streptavidin conjugated to FITC. Apoptotic cells within the same microscopic field were viewed and photographed by phase contrast and by fluorescence. Using the FITC filter, early apoptotic cells (positive for Annexin V-Biotin-FITC staining) appear bright green. C. Western blot analysis of poly (ADP-ribose) polymerase (PARP) protein, caspase 9, BAK, Bcl-2 and control β actin with increased doses of SFN. Native and cleaved PARP, pro and active caspases are indicated by arrows.

Inhibition of cell cycle progression in tumor cells may be associated with a concomitant activation of cell death pathways such as apoptosis. We, therefore, examined the contribution of apoptosis in SFN-treated MDAH-2774 cells. Apoptosis induces changes on the cell surface, which results in translocation of phosphatidylserine (PS) from the inner layer of the plasma membrane to the outer layer [18,19]. Annexin V is a Ca^2+ ^-dependent protein with high affinity for PS [19] that was used to assess apoptotic cells. Flow-cytometric analysis was performed to quantify apoptotic changes after treatment with SFN. Control and treated cells were prepared for bivariate analysis using annexin V stain that detects cells undergoing apoptosis and propidium iodide (PI) to detect nonviable cells. The number of pro-apoptotic cells after 48 h of 10 μM SFN treatment was substantially higher as compared with control. We observed that the ratio of apoptotic cells increased with the increased dose of SFN treatment: approximately 8% (5 μM treatment) and 20% (10 μM treatment) (Fig. 2A) of the cells over 48-hour period. Further we analyzed cells by immunofluorescence staining with Annexin V for apoptosis after SFN treatment. After 48 hr exposure to 10 μM SFN, 60% of the cells stained positive for annexin V, whereas untreated were less than 5% annexin V positive (Fig. 2B). As shown in Fig. 2C, western blotting analysis revealed the activation of PARP and of caspases-9 from counterpart pro-caspase. Further we document a dose-dependent increase in pro-apoptotic BAK and decrease in the expression of the anti-apoptotic protein Bcl-2.

### SFN inhibits S phase entry of MDAH-2774 cells in cultures

**Figure 3:**
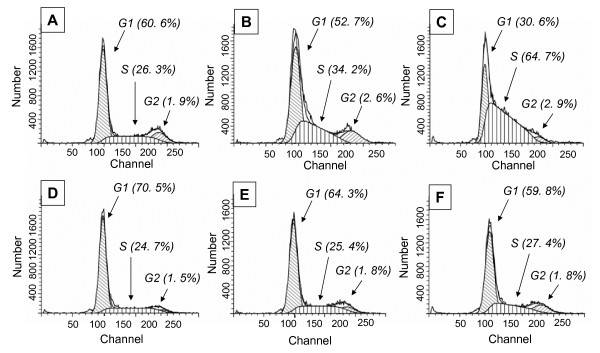
**Effect of SFN on DNA synthesis of MDAH-2774 cells**. ***A***. Cells were seeded at 10^6 ^cells and grown serum-free medium for 24 hr for cell cycle synchronization. Cells were then treated with either vehicle DMSO (i, ii and iii) or 10 μM SFN (iv, v and vi) for 0 hours (i and iv), 24 hours (ii and v) and 48 hours (iii and vi), respectively. Cells were fixed, stained and flow cytometric analysis was conducted using a cell cycle phase determination kit (Cayman chemical company, Ann Arbor, MI). **B**. Tabular representation of the % changes in the cell cycle phases. * Metaphase not reported.

In order to determine the causes of the growth inhibition observed by cell proliferation assays, we assessed the cell cycle progression of the cells in the presence of SFN. Serum starved MDAH-2774 cells were treated with either vehicle or 10 μM SFN for 12 or 24 hrs with complete growth medium. The cells were then washed, fixed, and cell cycle phase determination was performed utilizing flow cytometry and a cell cycle phase determination kit. The results demonstrated that SFN induces G1 arrest in a time-dependent manner (Fig. 3D, E and 3F) in comparison with control cells (Fig. 3A, B and 3C). There is a negligible increase of S phase cells after 12 hrs (0.7%) and 24 hrs (2.7%) with SFN treatment compared with 40% of cells in S phase for control cells at 24 hrs. Further we observed an overall increase in the cells at G1 phase of the cell cycle with SFN treatment.

### SFN induced modulation of cell cycle regulatory genes

**Figure 4:**
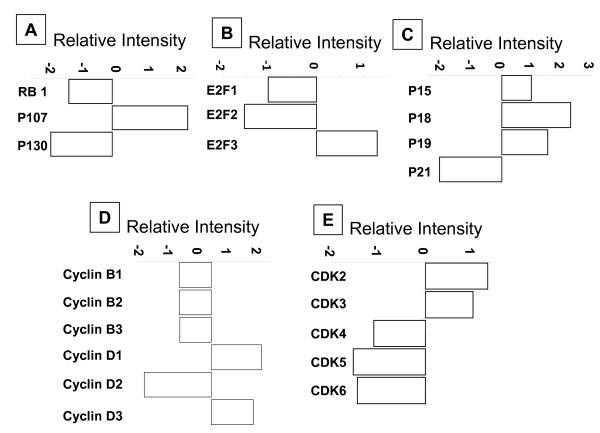
**Gene array analysis of indicated cell cycle regulatory proteins**. MDAH-2774 cells were treated with 10 μM SFN for 48 h were harvested and total RNA was isolated to generate cRNA and was hybridized to Whole Human Genome (G4112A) arrays (Agilent Technologies, Santa Clara, CA) according to the manufacturer's instructions. A. Retinoblastoma family of proteins; B. E2F transcription factors; C. Cyclin dependent kinase inhibitory proteins (CDKIs); D. Cyclins; E. Cyclin dependent kinases.

Since we observed cell cycle inhibition with SFN treatment at G0/G1 phase of the cells, we evaluated genes that influence the cell cycle progression into S phase. Under phosphorylated tumor suppressor retinoblastoma proteins (RB, p107 and p130) sequesters cell cycle promoting E2F-1 transcription factors. Gene expression analysis of RB proteins revealed a 1.5 and 2.0 fold decrease of RB and p130, respectively; as well as, a 2 folds increase in p107 levels (Fig. 4A). The E2F family of proteins, E2F-1, 2 and 3 which interact with RB, demonstrated 1.0 and 1.5 fold reduction in the expression levels of E2F-1 and 2, respectively, and 1.0 fold increase in the levels of E2F-3 (Fig. 4B). Cyclins and cyclin dependent kinases (CDKs) and their inhibitors exhibit distinct expression patterns, which contribute to the temporal coordination of each event in cell cycle progression. SFN treatment resulted in the decreased expression of G1 phase cyclins and CDKs while increasing the expression of cyclin dependent kinase inhibitors (CKIs), which bind and inhibit the activity of cyclin/Cdk complexes and negatively regulate cell cycle progression (Fig. 4C, 4D &4E).

### Inhibition of the phosphorylation of Retinoblastoma protein (RB) and protection of RB-E2F-1 complex by SFN

**Figure 5:**
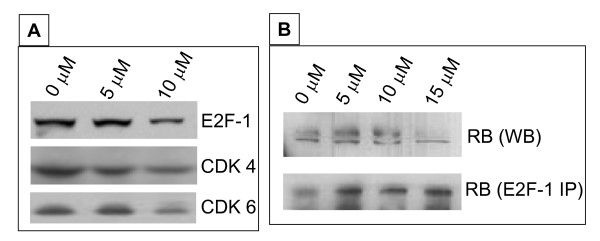
**Protein expression analysis of cell cycle regulatory proteins RB-E2F-1 interactions**: A. Approximately 10 μg of protein extracts of control and SFN treated MDAH-2774 cells were resolved by SDS-PAGE, transferred to nitrocellulose membrane; and probed with E2F-1, CDK-4 and CDK-6 as indicated. β actin was used as a loading control. B. Western blot analysis of SFN treated MDAH-2774 cells (upper panel). SFN treated MDAH-2774 cell lysates were immuno-precipitated with E2F-1 polyclonal antibody and probed with RB monoclonal antibody (Lower panel). P-RB: hyper phosphorylated retinoblastoma protein; RB: Under phosphorylated retinoblastoma protein.

Under-phosphorylated active retinoblastoma protein (RB) family of proteins, RB, p107 and p130 tumor suppressor proteins control cell cycle progression through the late G1 phase to the S phase by inhibiting E2F family of transcription factors [15,20,21]. We observed progressive conversion of phosphorylated RB to non-phosphorylated RB after 48 hrs with when MDAH-2774 cells were treated with 5 to 15 μM SFN treatment (Fig. 5B upper panel). Further E2F-1 is known to be elevated in many tumor cell lines and responsible for the expression of S phase genes that drives cell cycle progression. We observed decrease in the E2F-1 protein levels with SFN treatment corroborating cell cycle analysis results of decreased number of cells in S phase (Fig. 5A). Cyclins and cyclin-dependent kinases (CDKs) regulate the activity of RB by phosphorylation resulting in control of progression through G1 [16]. Since we observed elevated levels of under-phosphorylated RB, as expected, we observed lower levels of CDK4 and CDK6, the proteins responsible phosphorylation of RB in response to SFN in dose dependent manner (Fig. 5A). To assess if SFN treatment increases the RB-E2F-1 interaction resulting low levels of E2F-1, we conducted immunoprecipitation of cell extracts with E2F-1 polyclonal antibodies and probed with RB monoclonal antibodies. We observed a dose dependent increase in the RB levels in E2F-1 immunoprecipitates (Fig. 5B).

### SFN inhibits cell motility and invasiveness

**Figure 6:**
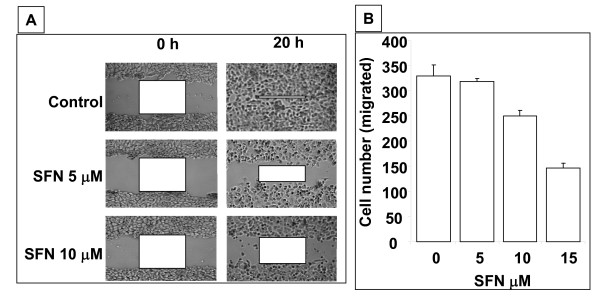
**Wound healing and cell migration assays**. A. Cell motility in wound healing assay. A uniform scratch was made in 80% confluent monolayer culture of MDAH-2774 cells and the extent of closure was monitored under phase-contrast microscopy as indicated and photographed. Representative images of two independent experiments done in duplicate are shown. B. Chemotactic migration of MDAH-2774 cells through membrane in Boyden chamber. Logarithmically growing cells were trypsinized and seeded in Boyden chambers and treated with various concentrations of SFN as described in methods. Migrated cells through the membrane were stained and counted with microscopy. Results of three independent experiments were plotted.

Cell motility following wound generation showed a greater cell migration in control cells compared with SFN treated cells. After 20 hrs, we observed almost complete closure of the wound in control cells which was inhibited by 50% and 75% by 5 μM and 10 μM SFN, respectively (Fig 6A). Cell migration was also inhibited when treated with SFN in a modified Boyden chamber. A progressive decrease in the cell migration through membrane with the SFN treatment from up to 15 μM was observed (Fig. 6B).

## Discussion

SFN mediating cell growth arrest has been documented in colon, prostate and several other cancers [6-8]. Chaudhuri et al, recently demonstrated that SFN inhibits the growth of the ovarian cancer cells and the inhibition of the AKT pathway is one of the upstream molecular events [9]. In this report we investigate downstream molecular mechanisms at the level of cell cycle control in the nucleus.

Combination therapy with paclitaxel is known to increase overall survival [3], but contributes to the development of resistance phenotype resulting in eventual relapse of the disease [22]. Unlike traditional chemotherapy drugs, natural therapeutics like SFN may not contribute to the development of chemo-resistant phenotype. Our results indicate a synergistic effect of cell death when SFN was used in combination with paclitaxel (Fig. 1D).

SFN treatment resulted in cleavage of Poly (ADP-ribose) polymerase-1 (PARP-1) in dose dependent manner indicating the induction of apoptosis that was further confirmed by annexin V staining. Induction of apoptosis by SFN in different cellular systems is associated with Bax protein expression [23]. Similarly the present study indicates a dose-dependent inhibition of anti-apoptotic protein Bcl-2 and concomitant increase in the expression of the pro-apoptotic protein Bak protein.

Phosphorylated RB cannot interact with E2F-1, thus leaving large pool of free E2F-1 transcription factors driving the G1/S cell cycle transition. E2F-1 has been shown to have growth promoting activity in EOC and is over expressed in roughly half of the ovarian cancers [24]. Similarly, studies with histopathology grade 2 ovarian cancers demonstrated that transcription factors E2F-1 and E2F-2 play a critical role in promoting tumor metastasis [25]. In our study, gene expression profiling has demonstrated down regulation of E2F-1 and 2 but not 3 [25]. Although we observed down regulation of RB transcript with SFN treatment in gene profile analysis, over 90% of the translated protein product is in the active, under phosphorylated form. This supports our hypothesis that the appearance of under phosphorylated RB with SFN treatment results in the inhibition of cell cycle progression by the reduction in free E2F-1. Co-immunoprecipitation experiments also demonstrated the increased expression of the RB-E2F-1 complex with SFN treatment potentially adding to the reduction of E2F-1 levels.

Gene profile analysis showed down regulation of these CDKs with SFN treatment corroborating the under phosphorylated status of RB. Although SFN treatment is known to causes induction of p21 in prostate cancer [26], in our gene profile analysis, we observed a twofold decrease in p21. It is possible that in the case of ovarian cancers, CDK inhibition works through the induction of p15, p18 and p19 following up regulation with SFN treatment.

Invasion followed by degradation of basal membrane is hallmark of tumor metastasis where proliferating tumor cells infiltrate into other tissues [27]. Wound healing assays and Boyden chamber assays provide evidence that the cell motility and invasiveness are inhibited by SFN. These findings suggested that SFN has very good potential for use in the treatment against invasion and metastasis of EOC.

## Conclusions

SFN induces growth arrest and apoptosis in EOC cells by inhibiting RB phosphorylation and reduction in the levels of free E2F-1. In summary, we have provided evidence that SFN suppresses growth of EOC cells *in vitro *by contributing to the modulation of cell cycle regulatory proteins and by increasing the apoptosis. These effects may be correlated to the observed inhibition of cell migration. These observations highlight the possibility that SFN may be a good candidate for combination therapy of EOC with paclitaxel.

## List of Abbreviations

SFN: Sulforaphane; EOC: Epithelial Ovarian Cancer; RB: Retinoblastoma.

## Competing interests

The authors declare that they have no competing interests.

## Authors' contributions

All authors read and approved the final manuscript. RBB designed the work and wrote the initial drafts of the manuscript. MP and CS supervised cell cycle analyses and contributed to data interpretation. CSB and SK carried out growth curves and paclitaxel combination experiments. JP and SC carried out RB-E2F-1 co-immunoprecipitation experiments. JS and SS performed most of the western blot analysis. JP carried out the western blots of caspase substrate cleavage. MS made original observations leading to this work and contributed to the critical revision of the manuscript. AMQ and MH conducted flow cytometry experiments and helped generate graphs from flow cytometry analysis. MBL provided valuable reagents and contributed to the critical revision of the manuscript. RM, AS and RBP contributed to the experimental design and critical revision of the manuscript. DWW contributed to the conception and design of the entire study and the final editing of the manuscript.
